# INCREASING THE REPERTOIRE FOR DEPRESSION CARE: PEER SUPPORT AS DEPRESSION CARE FOR VULNERABLE OLDER ADULTS

**DOI:** 10.1093/geroni/igad104.1801

**Published:** 2023-12-21

**Authors:** Jinhui Joo, Melissa Davey-Rothwell, Namkee Choi, Joseph Gallo, Alice Xie

**Affiliations:** Masschusetts General Hospital, Boston, Massachusetts, United States; Johns Hopkins University, Baltimore, Maryland, United States; University of Texas, Austin, Texas, United States; Johns Hopkins University, Baltimore, Maryland, United States; Massachusetts General Hospital, Boston, Massachusetts, United States

## Abstract

Despite facing greater risks for poorer health, low-income White and BIPOC older adults underutilize mental health services even when they have indicated need. Increasing the repertoire for depression care that is community-based and uses paraprofessionals has potential to increase access and engagement. We are testing the effectiveness of a peer support intervention called Peer Enhanced Depression Care (PEERS) which is an 8-week community-based intervention that uses trained peer mentors to deliver emotional, appraisal and informational support in addition to encouraging self-care skills to depressed low-income white and BIPOC older adults. Enrolled participants are randomized to either the peer support intervention (PEERS) or to the social interaction control and followed for 12 months. The primary outcome is depression and secondary outcomes include engagement, mental health service use, and social, emotional, and physical functioning. Challenges related to the onset of the COVID-19 pandemic and social isolation required a shift from recruitment initially focused on the health care system to community-based organizations serving older adults. Required contactless recruitment strategies eg. flyers and newspaper ads, led to self-referral of community-dwelling older adults to the study. Challenges to participant enrollment included barriers related to communication, stigma related to help-seeking, distrust and unfamiliarity with research. Recruitment of peer mentors was facilitated by a robust infrastructure supporting the employment of the peer support workforce. Continued PM supervision after initial training, review of skills and evaluation of performance were important in maintaining quality and fidelity to the intervention.

